
JOURNAL INFORMATION
==============================
Journal ID: Wirtschaftsdienst

Wirtschaftsdienst

EISSN: 1613-978X

ARTICLE INFORMATION
==============================
PMCID: PMC8933207
DOI: 10.1007/s10273-022-3132-5
Article ID: 3132
Article version: 1
Subjects: Zeitgespräch

Verbraucherkredit als Daseinsvorsorge?

Consumer Credit as a Provision of General Interest?

Domurath Irina
Central University of Chile, Oranienburger Straße 45, 10117 Berlin, Deutschland
Electronic publication date: 2022 Mar 19
Print publication date: 2022
Volume: 102
Issue: 3
First page: 189
Last page: 192
Copyright: © Der/die Autor:in 2022
License: Open Access: Dieser Artikel wird unter der Creative Commons Namensnennung 4.0 International Lizenz veröffentlicht (https://creativecommons.org/licenses/by/4.0/deed.de).
Open Access wird durch die ZBW — Leibniz-Informationszentrum Wirtschaft gefördert.
License URL: https://creativecommons.org/licenses/by/4.0/

Keywords: G51, G53
issue-copyright-statement: © The Author(s) 2022

==============================

Die COVID-19-Pandemie hat einmal mehr gezeigt: in Krisenzeiten, sind es oft die finanziell Schwachen, die besonders von gesamtwirtschaftlichen Einbrüchen betroffen sind. Wie schon in der Finanzkrise bietet sich jetzt eine Gelegenheit, die Rolle von Verbraucherkrediten in der heutigen Gesellschaft zu überdenken und Rechtsreformen anzustoßen, die diese Rolle angemessen widerspiegeln. Die Idee dieses Gedankenexperiments ist einfach: Verbraucherkredite sind aus der heutigen Gesellschaft nicht mehr wegzudenken. Er ist, wie andere systemrelevante Dienstleistungen (Telekommunikation, Wasser etc.), eine Dienstleistung der Daseinsvorsorge. Diese nehmen eine gesellschaftlich und sozial zentrale Stellung ein. Daher sollte auch die Überschuldung anders behandelt werden und nicht dem „Geld hat man zu haben“-Grundsatz unterstellt werden. Der Rechtsrahmen für den Verbraucherkredit sollte die Systemrelevanz von Krediten widerspiegeln.

## Systemrelevanz des Verbraucherkredits

Der Verbraucherkredit kann schon allein deswegen als systemrelevant – also: gesellschaftlich unverzichtbar – angesehen werden, weil er (systemrelevanten) Konsum ermöglicht. Konsum ist heutzutage ein Grundpfeiler für Wirtschaftswachstum. Dies wurde auch zuletzt in der COVID-19-Pandemie deutlich. So hat die Bundesregierung der Autoindustrie mit 3 Mrd. Euro für unter anderem konsumgestützte Maßnahmen unter die Arme gegriffen: 1 Mrd. davon wurde für Kaufprämien für Elektroautos eingesetzt. Mit diesen Maßnahmen sollen Verbraucher:innen dazu angehalten werden, neue Autos zu erwerben. Da der private Autokauf oftmals mithilfe von Verbraucherkrediten finanziert wird, zeigt dies, dass Verbraucher:innen und – im Zweifel auch durch Kredit finanzierter – Konsum Grundpfeiler für die wirtschaftliche Erholung darstellen.

Darüber hinaus ist der Verbraucherkredit für eine moderne Geldpolitik unabdingbar. Heutzutage wird die Mehrheit des Geldes durch kommerzielle Banken bei der Kreditvergabe erschaffen (McLeay et al., 2014). Damit ist der Verbraucherkredit mit der Zentralbankpolitik und den Mechanismen globaler Finanzmärkte verknüpft. Dies wurde in der Finanzkrise 2008 deutlich: eine Krise auf dem zunächst lokal begrenzten Immobilienkreditmarkt in den USA weitete sich durch systemische Verknüpfung weltweit aus.

Zuletzt ergibt sich die Systemrelevanz von Verbraucherkrediten insbesondere aus seiner Funktion im „umgebauten“ Sozialstaat. Der Verbraucherkredit ist heutzutage deswegen systemrelevant, weil der Zugang zu Krediten mehr und mehr Wohlfahrtsdienstleistungen ersetzt hat. Prasad (2012; 2019) bezeichnet dies als den „credit-welfare trade-off“. Damit beschreibt sie die Entwicklung der vergangenen Jahrzehnte, in denen sich Wohlfahrtstaaten zunehmend von der Garantie der Wohlfahrt verabschiedet und im Gegenzug vermehrt und vereinfacht Zugang zu Verbraucherkrediten gewährt haben (auch indirekt durch die Neuregulierung der Finanzmärkte). Diese Entwicklung ist anhand der invertierten Beziehung zwischen öffentlichen Fürsorgeleistungen und dem Ausbau von Verbraucherkrediten sichtbar: in höher entwickelten Sozialstaaten ist die Nachfrage nach Verbraucherkrediten demgemäß geringer als in weniger entwickelten Sozialstaaten (Prasad, 2012). In letzteren gibt der private Kredit Verbraucher:innen die Möglichkeit, ihren Konsum, einschließlich der Basisgüter, zu finanzieren. Damit werden also öffentliche Ausgaben durch private Verschuldung ersetzt (Pierson, 1994; Castles et al., 2010; Nullmeier und Kaufmann, 2010). Kredite erlauben unter anderem den Erwerb von Verbrauchsgütern und Leistungen, die sonst vom Staat erbracht würden.

Auch in Deutschland ist diese Entwicklung sichtbar: die politische Förderung von Sparverhalten erodierte, quasi-öffentliche Darlehen und Verbraucherkredite wurden verbreitet angeboten und der Sozialstaat ist in den Bereichen Rente, Bildung und Gesundheitsvorsorge weniger aktiv (Mertens, 2017). Der deutsche Sozialstaat hat sich als „aktivierender Wohlfahrtsstaat“ „restrukturiert“. Dies bedeutet nichts anderes, als dass der Staat seine Funktion nicht darin sieht, Bürger:innen direkt Wohlfahrt zu sichern, sondern sie dabei zu unterstützen, sich selbst um ihre Wohlfahrt zu kümmern. Schröders Agenda 2010 war das Paradebeispiel für diesen Umbau des Sozialstaates: Durch die sogenannte Flexibilisierung des Arbeitsmarktes (die bezüglich der Möglichkeit zur befristeten Beschäftigung für über 50-Jährige vom EuGH für als diskriminierend und damit EU-rechtswidrig befunden wurde), die Zusammenlegung des Arbeitslosengeldes mit der Sozialhilfe (im Endeffekt eine Kürzung des Arbeitslosengeldes), die Erhöhung des Rentenalters etc. ist Deutschland der Third Way-Politik von Blair's Labour Partei in Großbritannien gefolgt (Blair und Schröder, 1998, Schröder, 2003). Da also Verbraucherkredite für Wirtschaftswachstum, Geldpolitik und Sozialpolitik unentbehrlich sind, sollten sie auch als systemrelevant angesehen werden.

## Strukturelle Macht der Banken

Die Systemrelevanz des Verbraucherkredits bringt auch Banken in eine systemrelevante Stellung. Sie haben eine strukturelle Macht, weil sie Macht über Kreditkapazitäten haben: die Macht zur Kreditvergabe bedeutet auch, anderen die Möglichkeit des „Kauf-heute-bezahl-morgen" einzuräumen oder zu verwehren und damit über die Möglichkeit zu entscheiden, wer heute Kaufkraft hat, um Märkte anzukurbeln (Strange, 2015). Diese Macht ist strukturell, weil sie in das globale System von Geld- und Wertschöpfung eingebettet ist, indem kommerzielle Banken Geld durch Kreditvergabe generieren (McLeay et al., 2014). Dieses System legt eine Struktur dar, die auf relativ stabile Art und Weise ein bestimmtes Verhalten sowohl von Banken als auch ihren Kund:innen verlangt. Dazu gehört nicht zuletzt das Verbraucherwohlverhalten, um eine gute Bonität zu erreichen. Kreditwürdigkeitsprüfungen des § 505a BGB, die Banken seit Jahrzehnten routinemäßig durchführen, haben einen maßgeblichen Einfluss auf die (Nicht-)Gewährung von Krediten. In dem modernen wirtschaftspolitischen System, in dem der Verbraucherkredit eine wohlfahrtssichernde Funktion einnimmt, ist das Ergebnis der Bonitätsprüfung damit auch eine indirekte Entscheidung über den Zugang zu und die Verteilung von Wohlfahrt.

Gleichzeitig wird Banken damit auch eine quasi-politische und -legislative Funktion zuteil. Dies ergibt sich zum einem aus der Verwendung von Standardkreditverträgen und AGB, die die Verbraucher:innen nicht verhandeln, und zum anderen aus ihrer sozialen Rolle, aus der sie die Verbraucher:innen zu bestimmten Verhalten bewegen können – wie beispielsweise durch die normativen Effekte von Kreditwürdigkeitsprüfungen. Ausgestattet mit quasi-öffentlicher normativer Autorität stellen Banken Regeln für Wohlverhalten in Kreditbeziehungen auf und im „credit-welfare trade-off", damit auch für den Zugang zur Wohlfahrt.

## Parallelen zu Dienstleistungen der Daseinsvorsorge

Unter Zugrundelegung eines Verständnisses von Verbraucherkrediten als systemrelevante Dienstleistung, die von strukturell mächtigen Banken angeboten werden, kann durchaus argumentiert werden, dass Banken – über ihre bereits bestehenden Informations- und Sorgfaltspflichten der §§ 491ff. BGB hinaus – besondere Pflichten auferlegt werden sollten. Die Rechtsinstitute der Dienstleistungen der Daseinsvorsorge sind als Vorbild besonders geeignet, weil sie einen Rechtsrahmen für unabdingbare Leistungen schaffen und damit der Systemrelevanz des Verbraucherkredits Rechnung tragen. Dienstleistungen zur Daseinsvorsorge sind Telekommunikation, Energie- und Wasserversorgung, Transporte und Postdienste, die für die gesellschaftliche Integration unentbehrlich sind. Ihr Rechtsrahmen ist in Bezug auf die Verbraucher:innen von Kontrahierungszwang und Erschwinglichkeitskriterien (sowie Marktaufsicht) geprägt.

Allerdings kommt ein Kontrahierungszwang für einen Verbraucherkredit nicht infrage, weil es dem Interesse der nicht kreditwürdigen Verbraucher:innen entgegensteht, eine Kreditverpflichtung einzugehen, die wahrscheinlich nicht erfüllt werden kann. Damit würden sich die finanziellen Probleme der Verbraucher:innen nur verschieben und vervielfachen. Darunter würden erneut besonders die Einkommensschwachen und Armen leiden. Es wäre also für niemanden sinnvoll, Kreditinstitute zu verpflichten, Verbraucher:innen unabhängig von jeglicher Bonität Kredit zu gewähren.

Damit wird Erschwinglichkeit zum maßgebenden Parameter. Eine Grundversorgung mit Verbraucherkrediten ist nur dann sinnvoll und machbar, wenn sie auch erschwinglich sind und die finanziellen Probleme von Verbraucher:innen nicht noch verstärkt. Für den Verbraucherkredit würde Erschwinglichkeit bedeuten, dass die Bank einkommensschwachen Verbraucher:innen nur bestimmte Kredite anbieten darf. Wenn beispielsweise der Gesamtbetrag des geforderten Kredits so hoch ist, dass auf Basis ihres Einkommens absehbar ist, dass die Verbraucher:innen ihren monatlichen Zahlungen nicht dauerhaft nachkommen können, sollten der Kreditgeber dazu verpflichtet sein, den Gesamtbetrag zu reduzieren, Kredite mit nur kurzen Laufzeiten anzubieten oder weniger bzw. keine Zinsen zu verlangen. Auf diese Weise würde die finanzielle Belastung der Rückzahlung für Verbraucher:innen gesenkt und die Rückzahlungschance erhöht. Ein solches Vorgehen würde es außerdem erlauben, dass Verbraucher:innen mit schlechter Bonität nicht per se von der Kreditvergabe ausgeschlossen würden. Im Gesetz vorgeschriebene Erschwinglichkeitskriterien für sämtliche Formen von Verbraucherkrediten könnten dazu beitragen, über die

Resultate von Bonitätsprüfungen hinaus sicherzustellen, dass Verbraucher:innen der Zugang zu sicheren Krediten gewährt wird. Damit würde ein Kompromiss geschlossen zwischen dem Schutz vor Überschuldung und dem Interesse, Verbraucher:innen möglichst Zugang zu Krediten zu gewähren, um *erstens* persönliche finanzielle Engpässe abzufedern und *zweitens* für die wirtschaftliche Erholung systemrelevanten Konsum zu ermöglichen.

## Neuverhandlung und Schuldenerlass

Über die Erschwinglichkeit des Verbraucherkredits als Daseinsvorsorge hinaus sollten außerdem die Möglichkeiten einer Neuverhandlung und eines Schuldenerlasses eruiert werden. Bezüglich der Neuverhandlung von Kreditkonditionen könnte eine weitere Ausnahme vom Grundsatz „pacta sunt servanda" eingeführt werden. Variationen des „rebus sic stantibus" werden insbesondere in Krisenzeiten immer wieder diskutiert (in der Finanzkrise: Domurath, 2017; für die Coronakrise: Twigg-Flesner et al., 2020). Es wird vorgeschlagen bei social oder societal force majeure (Wilhelmsson, 1990) eine Neuverhandlung des Vertrages zu bewirken. Auch das European Law Institute in Wien hat 15 Grundsätze für die Coronakrise veröffentlicht, darunter zwei Grundsätze, die sich mit solchen unvorhergesehenen Umständen beschäftigen. In Deutschland könnte § 313 BGB auf Fälle angewandt werden, bei denen die Einkommensquelle aufgrund der Pandemie verloren gegangen ist. Keine Vertragspartei muss das Risiko hierfür tragen.

Für die Anpassung von Kreditkonditionen spricht auch die lange Dauer von Kreditverträgen. Reifner plädiert schon seit Jahren für die rechtliche Einordnung von Kreditverträgen als Dauerschuldverhältnisse – lebenslange Verträge (Reifner, 2015; auch: Pulgar, 2014). Die Verbraucherzentrale Bundesverband (VZBV) hat sich auch für einen „dynamischen" Blick auf die Kreditbeziehung ausgesprochen; und spricht in einem Papier zum „produktiven Kredit" von der „produktiven Anpassung der Kreditkonditionen" (Roggemann et al., 2021).

Grundsätzlich führt § 313 Abs. 1 BGB zu einem Anspruch auf Vertragsanpassung (Lüttrighaus, 2013; Thole, 2014). Ist eine Vertragsanpassung nicht möglich, bestimmt § 313 Abs. 3 BGB für Dauerschuldverhältnisse das Recht auf Kündigung. Dies ist für Verbraucher:innen in Zahlungsschwierigkeiten nicht hilfreich, da Rechtsfolge der Kündigung die Fälligstellung des Kredites ist, den die Verbraucher:innen ja gerade nicht bedienen können. Es könnte daher eruiert werden, die Verhältnismäßigkeitsprüfung des § 489 BGB – in Anlehnung an § 19 StromGV –, zugunsten der Verbraucherschuldner zu verbessern. Im Moment kann die Kündigung des Darlehensvertrages durch den Gläubiger bei Schuldnerverzug gemäß § 489 BGB nur dann erfolgen, wenn die Verbraucher:innen mit mindestens zwei aufeinanderfolgenden Raten ganz oder teilweise mit mindestens 10 % in Verzug sind, bei Darlehen von über drei Jahren mit mindestens 5 % der Darlehenssumme. Es ist zu erwägen, die Zahl der Raten, mit denen Verbraucher:innen in Verzug sein müssen, um das Kündigungsrecht des Gläubigers auszulösen, zu erhöhen, da in Pandemiezeiten zu erwarten ist, dass Zahlungsschwierigkeiten über zwei Monate hinaus andauern. So könnte Verbraucher:innen die Anhäufung von Verzugszinsen und auch Rechtsstreitigkeiten erspart bleiben.

Aus Verbraucherschutzsicht ist außerdem zu erwägen, Schuldenerlass auch außerhalb des Insolvenzverfahrens zu gewähren. Schuldenerlass kommt insbesondere bei kleinen Verbraucherkrediten infrage, die die Verbraucher:innen zur Deckung wiederkehrender Zahlungen (Wohnraummiete, Strom etc.) aufnehmen. Dies sollte insbesondere gelten, wenn der Kredit zur Tilgung von Rechnungen für weitere Leistungen der Daseinsvorsorge benutzt wird (Wasserversorgung, Telekommunikation, Energie; auch Wohnraum könnte hierunter fallen).

Laut European Law Institute (ELI) sollten als Grund für den Schuldenerlass Public-policy-Erwägungen ausreichen (ELI Grundsatz 12 Absatz 3, 1. Alternative). Eine solche Public-policy-Erwägung ist mithin die Vermeidung von weit verbreiteter Verbraucherinsolvenz, die sich nicht nur gesamtwirtschaftlich auswirken könnte, sondern auch auf Verbraucher:innen, deren finanzielle Möglichkeiten auf Jahre hin eingeschränkt sein könnten. Auch das VZBV-Papier zum produktiven Kredit erkennt an, dass private Überschuldung ein öffentliches Problem ist (Roggemann et al., 2021).

Schuldenerlass ist außerdem Moratorien (wie z. B. der temporären Aussetzung der Insolvenzantragspflicht in Deutschland) vorzuziehen, weil letztere die finanziellen Probleme von Verbraucher:innen und Selbständigen nicht lösen, sondern deren Auflösung nur aufschieben. Dahinter steht freilich der Gedanke, dass die Wirtschaft irgendwann wieder anspringt und sich die Verbraucher:innen wieder in eine bessere finanzielle Position bringen können (Twigg-Flesner et al., 2020). Allerdings übersieht dieser Ansatz, dass sich in der Zwischenzeit die Probleme noch verstärken können und Verbraucher:innen oftmals noch weitere Schulden anhäufen.

## Schlussbemerkung

Das Gedankenexperiment zeigt, dass es durchaus politischen und juristischen Handlungsspielraum gibt, um den systemrelevanten Verbraucherkredit adäquat rechtlich zu verankern. Dies kann dazu beitragen, die Verbraucherüberschuldung in Krisenzeiten abzumildern. Nun kann man die Charakterisierung von Verbraucherkrediten als systemrelevant von ideologischer Seite durchaus kritisch sehen. Selbstverständlich basiert diese Einordnung auf der politischen Akzeptanz der Third Way-Sozialpolitik, in der die Einzelnen und ihre Möglichkeiten und Freiheiten, sich möglichst ohne staatliche Hilfe das Leben zu organisieren, im Mittelpunkt stehen. In diesem Beitrag sollte es eher darum gehen, Verbraucherkredite in der beschriebenen Politikökonomie – die man nun für gut oder schlecht erachten kann – für Verbraucher:innen sicherer zu machen. Und damit auch für die Wirtschaft.

**Title:** ***Consumer Credit as a Provision of General Interest?***

The COVID-19 pandemic has shown once again that it is often the poor who suffer from economic downturn in times of crisis. As was the case in the financial crisis of 2008, we now have an opportunity to rethink the role of consumer credit in society and design and create a legal framework that adequately reflects that role. The following thought experiment hopes to give impetus to legal reforms.

Dr. Irina Domurath ist Associate Professor of Law an der Universidad Central de Chile.


Literatur

Blair, T. und G. Schröder (1998), Europe: the Third Way/die Neue Mitte, Friedrich-Ebert-Stiftung, Friedrich-Ebert Foundation South Africa Working Documents, 2.
Castles, F. G., S. Leibfried, J. Lewis, H. Obinger und P. Pierson (2010), Introduction, in F. G. Castles et al. (Hrsg.), Oxford Handbook of the Welfare State, Oxford University Press, 8 ff.
Domurath, I. (2017), Consumer Vulnerability and Welfare in Mortgage Contracts, Hart.
Lüttringhaus J D Verhandlungspflichten bei Störung der Geschäftsgrundlage: Ein Beitrag zur Dogmatik und Durchsetzung von Anpassungsanspruch und Verhandlungspflichten nach § 313 Abs. 1 BGB Archiv für die civilistische Praxis 2013 213 2 266 298 10.1628/000389913X13607714432081
McLeay, M., A. Radia, und R. Thomas (2014), Money creation in the modern economy, Quarterly Bulletin, Q1, Bank of England.
Mertens D Borrowing for social security? Credit, asset-based welfare and the decline of the German savings regime Journal of European Social Policy 2017 27 5 474 490 10.1177/0958928717717658
Nullmeier F Kaufmann F X Castles F G Post-War Welfare State Development Oxford Handbook of the Welfare State 2010 Oxford Oxford University Press 81 ff
Pierson, P. (1994), Dismantling the Welfare State? Reagan, Thatcher, and the Politics of Retrenchment, Cambridge University Press.
Prasad, M. (2012), The Land of Too Much — American Abundance and the Paradox of Poverty, Harvard University Press.
Prasad, M. (2019), The Trade-Off between Social Insurance and Financialization: Is There a Better Way?, Niskanen Center Policy Essay.
Pulgar, J. (2014), A Contractual Approach to Over-Indebtedness: Rebus Sic Stantibus Instead of Bankruptcy, in Nogler und Reifner (Hrsg.), Life Time Contracts — Social long-term contracts in labour, tenancy and consumer credit law, Eleven Publishing.
Reifner, U. (2015), Thesen zur Dogmatik eines sozialen Nutzungsvertrages (Life-Time Contract), in K.-O. Knops et al. (Hrsg.), Zivilrecht im Wandel, Springer.
Roggemann, H., H. Klinger, A. Fandrich, N. Korff, S. Peters, U. Reifner und I. Größl (2021), Gutachten zum produktiven Kredit, Verbraucherzentrale Bundesverband, Institut für Finanzdienstleistungen.
Schröder, G. (2003), Regierungserklärung, 14. März 2003, Deutscher Bundestag, Plenarprotokoll, 15/32, Agenda 2010.
Strange, S. (2015), States and Markets, Bloomsbury.
Thole C Renaissance der Lehre von der Neuverhandlungspflicht bei § 313 BGB? JuristenZeitung (JZ) 2014 69 9 443 450 10.1628/002268814X13929732689749
Twigg-Flesner C Consumer Law and Policy Relating to Change of Circumstances Due to the COVID-19 Pandemic Journal of Consumer Policy 2020 43 437 450 10.1007/s10603-020-09463-z PMC7397452 32836591
Wilhelmsson T Social force majeure — a new concept in Nordic Consumer Law Journal of Consumer Policy 1990 13 1 14 10.1007/BF00411866
